# Music and the politics of famine: everyday discourses and shame for suffering

**DOI:** 10.1111/disa.12662

**Published:** 2024-10-23

**Authors:** Naomi Pendle, Abraham Diing Akoi

**Affiliations:** ^1^ University of Bath United Kingdom; ^2^ Firoz Lalji Institute for Africa London School of Economics United Kingdom; ^3^ University of Johannesburg South Africa; ^4^ Independent Researcher, Juba South Sudan

**Keywords:** famine, music, shame, songs, South Sudan

## Abstract

Understanding the politics of famine is crucial to understanding why famines still occur. A key part of this is how famine is remembered, understood, and discussed. This paper focuses on songs popular among communities that have recently experienced deadly famine. Contemporary famines almost always manifest in armed conflict contexts, where there is limited political freedom. Here, songs and music can be an important way to debate sensitive political issues. This paper focuses on the way that songs and music shape ‘regimes of truth’ around famine, and who is shamed and held accountable for associated suffering. It is based on long‐term ethnographic research, the recordings of famine‐related songs, and collaborative analysis in Jonglei and Warrap States (South Sudan) in 2021–24. The paper shows how songs can mock soldiers for their seizing of assets during times of hunger and how they can create familial shame for famine suffering, shifting responsibility away from the real causes to family members.

## 1 INTRODUCTION^1^

There is an urgent need to understand why famines persist, because of the human suffering that they cause, and because of what their persistence reveals about contemporary global power and politics. However, contemporary scholarship on famines is ‘precarious’, scant, and episodic (Rubin, 2019; Jaspars, 2022). For decades, there has been a scholarly consensus that famine is the result of political failure or design, as opposed to natural causes (de Waal, 1997a, 2018a; Deng, 2002; Watts, 2013; Jaspars, 2018; Kuol, 2021). Scholars have highlighted how global political economies have created states of near‐permanent famine (De Castro, 1952; Deng, 1998; Watts, 2013; Maxwell and Majid, 2016). At the same time, deadly famines in recent decades have occurred during armed conflict (de Waal, 2018a). Famine has had a beneficial function for some political and wartime strategies (Keen, 2008), with starvation used as a weapon of war and authoritarian governance (Howard‐Hassmann, 2016; de Waal, 2018a). In recognition of this, the United Nations (UN) Security Council asserted that starvation was a war crime and so, potentially, appropriate for international legal criminal accountability (de Waal, 1997a, 2018a; Edkins, 2007; Conley et al., 2022).

These accounts are crucial for recognising that famine is the product of war, and not something that is naturally occurring. Yet, this focus on national politics and international policy needs to be complemented by attention to the detailed political realities on the ground and in contexts where famine occurs. This is because these local contexts allow us both to see how international and national politics manifest in people's lives, and how people experiencing this might try to resist them.

There is an older but small and rich ethnographic literature on famine (Vaughan, 1987; Cliggett, 2005; de Waal, 1989). Historians have more recently produced contextually‐nuanced discussions of famine politics (Law‐Smith, 1989; Graziosi, 2004; de Zwarte, 2020; Corporaal and de Zwarte, 2022), showing the great potential of more in‐depth local scholarship. A recent special issue of the *Third World Quarterly* journal has focused on the social meaning of famine by discussing associated memories and memorials, and this has started to highlight the value of paying attention to the social and political dynamics of famine in contexts where it occurs (de Waal, 2024).

To help fill this gap concerning the social and political dynamics of famine in contexts where it occurs, this paper explores the politics of famine in two regions of South Sudan, Jonglei and Warrap States, where there have been episodic famine levels of hunger since the 1980s. We examine how discourses and the ‘regime of truth’ about famine disperse throughout the social body (in the words of Michel Foucault), and how these structures are reasserted, and potentially resisted, through music and songs. Leading contemporary scholarship on famine has concentrated largely on actors and institutions (Sen, 1981; de Waal, 2018a). Drawing on Foucauldian ideas, we recognise the need to look beyond actors and institutions and assess how power is dispersed through the social body, including in the ‘regime of truth’ about famine (Foucault, 1980)—the regime that stretches into everyday routines and people's very sense of self (Foucault, 1982). Such regimes are not invented but instead build on existing micro practices of power. Jaspars (2021) has brought the discussion of the ‘regime of truth’ to famine studies through her focus on humanitarian discourses and their regime.

We explore here the politics of music and its ‘regime of truth’ in relation to contemporary famine. The paper was inspired by our ethnographic observations that showed us that, in South Sudan, there are a significant number of songs about contemporary and past famines, and that many of these are commonly known and listened to. Among Dinka communities in Jonglei and Warrap States, songs are a major form of creative expression and history‐telling (Impey, 2007; Cormack, 2021). We find that songs are not only used to open up space for political discussion about famine, but also that they can shut down protests and silence potential advocates for change. While famines are caused by governments, warring parties, and actors in global political economies (Watts, 2013; de Waal, 2018a), songs can create space for subtle forms of political contestation regarding this politics (Raheja, 2017). Alternatively, songs can also place blame on families for famine‐related suffering, limiting their abilities to dispute the politics which caused these deadly events.

Our focus on music is important for two reasons. First, contemporary famines almost always occur during armed conflict, and warring parties during armed conflict often curtail freedom of speech and public debate. Famine is a politically‐sensitive topic, and explicit public debate and discussion is frequently restricted. At the same time, we can miss the politics and protest that are happening when we only look for certain familiar forms of political engagement, such as civil society statements, political party slogans, and newspaper reports (Mampilly, 2023). In contexts with different cultural and political histories, we see very different patterns of political expression. Music can be a form of political expression, including about sensitive topics (Nannyonga‐Tamusuza, 2017). Songs in South Sudan can be intentionally opaque (Cormack, 2021), which creates space for prohibited expression. Music can be a vital tool in negotiating differences and subtly challenging dominant discourses (Montero‐Diaz and Wood, 2021). Montero‐Diaz and Wood (2021, p. 183) highlight the need to understand why ‘people engage with music to negotiate suffering, survival, escape, belonging and forgiveness’. Music can play a powerful role in bearing witness to what has taken place (Impey, 2014). Songs can be socially‐ and politically‐embedded acts of citizenship (Impey, 2014). Raheja's (2017) work on famines in colonial India underlines how there were articulate critiques of ‘custom’ and the colonial state during famine in sung narratives composed by a variety of Indians living through these times.

Second, music is important to understand the politics of ordinary people, when it is created, performed, and listened to by ordinary people; hence, songs provide key insights into ordinary people's experiences and the politics of famine. For example, literature on historic famines has noted the use of songs among ordinary people to discuss memories of famine in contexts where famine occurred. For example, Gerk (2023) has used music to explore memories of Ireland's Great Famine (1845–52) among Irish Americans, and Falc'her‐Poyroux (2014) emphasises how music provides insights into famine from the perspective of ordinary people. At the same time, since the days of Plato, music has been thought of as a source of power, and governments and political parties have used it to try to shape identities and political understandings (Andersson, 1981; Saunders, 2000; Street, 2003). Music as performed and listened to by people in famine contexts can provide an insight into the politics at play.

Having described our research method, the paper next provides an outline of famines in Jonglei and Warrap States, and an introduction to music in these regions. The rest of the paper then critically considers the regimes of truth embedded in some songs about famine from these locales. We discuss three examples of groups shamed or blamed during famine in these songs. First, we debate how songs were able to create a political space in which to be critical of powerful military actors and highlight their immoral behaviour. Songs shamed soldiers for having violated moral norms around food and cattle that resulted in loss of life and dignity. The songs demand that soldiers also comply with local moral norms to avoid the shame of causing famine suffering. Second, we assess how many songs shame families for failing to keep their members free from famine suffering. We underscore how songs, like the law and other social mechanisms (Pendle, 2023), can support social networks that provide a way for people to survive. At the same time, the songs shaming family members silence the families of those who have suffered and died from famine and reduce the potential for grief‐related activism targeting the real causes of famine. Third, we analyse criticism of migration away from contexts of famine, another common theme, and consider some of the micro politics that encourage people to stay in famine settings, including extremely deadly ones.

## 2 METHODS

This paper is based on ethnographic and qualitative research in Warrap and Jonglei States. These regions were selected as they have experienced deadly and reoccurring famine, but this is not intended to imply that other parts of South Sudan have not also experienced similar hardships. Famine research has been limited by the inaccessibility of sites of famine, especially because contemporary famines are occurring in areas of protracted armed conflict (Rubin, 2019). This restricted access has prevented the qualitative and ethnographic research that is needed to improve our understanding of the social and political meanings of famine (de Waal, 2024). We were in a rare position to carry out this ethnographically‐informed research because we either have homes in areas where famine has occurred in living memory, or because we have researched in these spaces for more than a decade, providing us with the relationships and contextual awareness needed for this work. One of the authors, Abraham Diing Akoi, was born in Jonglei State and has lived there intermittently over the years, both in times of famine and times of plenty. Even when not living in Jonglei, he speaks to people there daily. From 2017, he has intermittently carried out ethnographic, autoethnographic, and qualitative research in the state. He drew on autoethnographic methods to shape the questions that we asked, and the themes that we explored, in this paper. The other author, Naomi Pendle, initially moved to Warrap State to work in a school and university and ended up carrying out multi‐year ethnographic research from 2009–13. She has continued to visit the state, speak to people there, and conduct research on a regular basis over the past decade.

This paper is informed by these longer‐term ethnographic insights and relationships. At the same time, our initial insights into famine and our awareness of famine songs have incidentally emerged through these years of ethnographic work. Therefore, to refine and develop our understanding, we conducted 20 (focused) qualitative interviews and song recordings in 2023 and 2024 in Jonglei and Warrap States. We focus here on the songs' lyrics and not on their performance—the songs were performed for us, and not necessarily presented in their usual social setting. Future research that pays attention to the context of their performance would be useful. After the initial recording of the songs, they were translated and both authors carried out their own thematic analysis of them before then comparing findings and collaboratively agreeing on prominent themes and findings. We validated this analysis in over‐the‐phone conversations with some of our research participants.

This paper does not provide a comprehensive review of songs about famine in these communities; however, it does provide some initial insights into local famine politics. We intentionally avoid including the transcripts of whole songs as we are concerned that this would allow the people that are being sung about to be identified and this would further deepen the shame that they produce (the shame that we critically explore here). The one song that we do talk about by name and in more depth is that by Wol Longar from Warrap State, about the 1988 famine. The reason for doing so is because of its popularity and the fact that it is common in people's discussions of famine. All of the songs were initially sung in Dinka but are included here as English translations.

## 3 FAMINES IN JONGLEI AND WARRAP STATES, SOUTH SUDAN

South Sudan^2^ has experienced decades of armed conflict and intermittent periods of ‘no war, no peace’. This has included violence by international merchants and colonial‐era officials, as well as civil wars after Sudan gained independence from the United Kingdom in 1956. Patterns of warfare in Sudan since the mid‐nineteenth century have encompassed, inter alia, the destruction of property and the capture of labour in ways that have significantly impacted on food security (Deng, 1999, pp. 22–40).

The armed conflicts since the 1980s have also involved significant periods of deadly famine. In 1982, Ananya II rebels, based in Unity State, neighbouring Warrap, rebelled against Khartoum. The following year, the Sudan People's Liberation Army (SPLA) also formed in opposition to the Sudanese government, initiating its rebellion from what is now Jonglei State, and came to dominate the uprising. People from Warrap State were key to the leadership and mobilisations of the SPLA. Over the following two decades, the Sudanese government and the SPLA used an array of patterns of violence, many of which targeted and killed civilians, and others which involved the widespread destruction of property and sources of food (Johnson, 2003). From the 1990s, divisions within the SPLA also prompted significant South–South violence, including in Jonglei and Warrap States. During the 1980s and 1990s, the Sudanese government used starvation as a deliberate tactic of war (de Waal, 1997b). Warring parties diverted and looted food aid, attacking civilians and their property (Rone, 1999). The conflict also prevented access to markets and preferable grazing and arable land, as well as interrupting historical patterns of intercommunal movement that previously had allowed families to access food in neighbouring communities that were less famine‐prone.

Mortality during some of these famines in the late 1980s and late 1990s has been incredibly high. Keen (1991, p. 151) describes the 1988 famine in Bahr el Ghazal (including in the area that is now Warrap State) as one of the deadliest ever recorded, with Médecins Sans Frontières recording a mortality rate of 7.1 per cent of the population per week in a migrant camp at El Meiram (West Kordofan State, adjacent to Bahr el Ghazal). Abraham Diing Akoi remembers, first hand, deaths from hunger in the early 1990s because of offensives and displacement in Jonglei State. In 1998, there was another, extremely deadly famine, known locally in Warrap State as the *makrup*—which, in Dinka, means ‘the hunger that covered the whole place’. As a result, there was nowhere left to which to migrate to find food; its full coverage removed a key survival strategy. Deng (2002, p. 30) calculated that excess mortality between April 1998 and January 1999 because of famine in Bahr el Ghazal was around 78,000.

In the 2000s, the SPLA and the Sudanese government negotiated the Comprehensive Peace Agreement (signed in 2005) that ended the war between them, but deep divisions between South Sudanese, as a consequence of the fighting, created a context in which violence and armed conflict in South Sudan escalated in subsequent years (Jok and Hutchinson, 1999; Johnson, 2003, 2014; Pendle, 2020). In December 2013, there were deadly battles in the capital, Juba, which then quickly spread to different regions across South Sudan (Pendle, 2020). Depriving communities of food and the means to survive has remained a wartime tactic ever since (Anei, de Waal, and Conley, 2019).

In 2017, the UN (2017) declared a famine in Unity State (South Sudan). However, famine has followed a different pattern in South Sudan since 2013, as compared to that in the 1980s and 1990s. Like much of the armed conflict since 2013, famine levels of hunger have materialised in relatively small pockets of territory. Much of this has not resulted in a famine declaration because the areas are too small to prompt one, even when famine levels prove deadly (Newton et al., 2021). The international famine declaration system requires that a large number of people are impacted (Maxwell et al., 2020).

Many of these small pockets of territory in Jonglei and Warrap States have experienced armed groups deliberately cutting off communities from markets and humanitarian aid, while also limiting their ability to grow their own crops (Anei, de Waal, and Conley, 2019). For many South Sudanese, therefore, famine is not only a memory from the 1980s and 1990s, but also is a recent experience or a contemporary fear.

## 4 MUSIC IN JONGLEI AND WARRAP STATES, SOUTH SUDAN

This research was conducted among Dinka‐speaking communities in Jonglei and Warrap States and so it focuses on songs in the Dinka language. Dinka songs have been described as a way to get to know the Dinka people (Deng, 1973; Impey, 2007; Cormack, 2021). In these contexts, songs are a major means of disseminating histories through the community and over generations, as well as being a record of popular and artistic expression (Cormack, 2021). Impey (2013) has described how the themes of Dinka songs pertain to historical memory, justice, conflict resolution, and civic engagement. Nonetheless, songs are an underutilised source of oral history (Impey, 2007). Importantly, songs do not just provide a narrative of the facts of history, but are an interpretation of history (Cormack, 2021). This creates a truth about the past that also informs how the truth about the present is interpreted.

Dinka songs take a variety of different forms and are sung by an assortment of different people (Deng, 1973). For example, there are women's songs, initiation songs, and ox songs (Deng, 1973), as well as sacred songs, communal songs sung at celebrations, such as weddings, and individually composed songs (Ryle, 1987). There are also different taxonomies of song, including *diɛt ke kër* (insulting and shaming songs), and other types of songs, such as *diɛt ke waak* (cathartic songs) (Impey, 2013, p. 198). Consequently, there are songs which intentionally seek to humiliate and make claims about who is responsible.

Songs among the Dinka often take on autobiographical forms (Impey, 2013). A particularly prominent form of song is the ox song. A man, as he enters the age of adulthood, will pay (usually in the form of a cow) a song composer to write a song for his ox (*muor*). This song ox (*muor waar* in Dinka Bor and *muor wec* in Dinka Rek) will already have been carefully nurtured and will often have been brought a bell and tassels for decoration. Its horns will also have been carefully shaped over time. When the man travels to the song composer for the ox song, the *muor waar* will travel with him, and the ox song will be sung to the animal. This ox displays the pride and status of the cattle herder.

Importantly for this paper, before the young man travels to the song composer, he must spend time speaking to family elders to learn his family history. His research will be shared with the composer to inform the song. These ox songs are thus usually a family‐made history of events and family members who have shaped what the family, and the young man, have become. Many of the songs that we draw on here are ox songs.

Dinka songs cover a range of topics, including famine, and debates. Kuol's (2021) work on famine among the Dinka has made use of various songs to illustrate his arguments. For instance, he cites lyrics by Atiam Thon, describing the collecting of wild fruits as a survival strategy. This Dinka poet in Gogrial (now part of Warrap State) critiques the loss of knowledge of fruit among those who ‘have been abandoned by Monyjang’ because they ‘turned their lives to the towns’ (Kuol, 2021, p. 77). According to the song, their lack of knowledge of local, rural foods puts them in danger. In addition, Kuol (2021, p. 41) cites a song by Mathuc Bol (also from Gogrial) that describes famine that kills: ‘The famine that has come with its knife to kill people. .. until it has blunted the spade whilst digging graves’. Mathuc Bol also used songs to critique humanitarian responses to famine and the lack of assistance (Kuol, 2021, p. 177).

In the context of our research, songs were composed both by local songwriters and by members of the public. They were also shared and sung by a wide collection of ordinary people, and increasingly on mobile telephones. For example, one of the songs we draw on is a particularly famous composition by Wol Longar in Warrap State. While we had the privilege of being able to interview and record Wol Longar himself, his song was also played to us by many research participants and friends, as they have his song on their phones.

## 5 WHAT REGIMES OF TRUTH HAVE BEEN CREATED BY FAMINE SONGS?

We move on now to explore some of the regimes of truth and discussions of blame and responsibility that appear in famine songs in Jonglei and Warrap States. This is not a comprehensive survey of famine songs, but the extracts included here are from songs that were being performed and listened to during our work, and that were shaping the everyday politics of famine in these places.

### 5.1 Using songs to shame soldiers

One famine song recorded by us focuses on shaming soldiers for famine and demands that they should also comply with the moral norms of the communities. This illustrates the space created by famine songs for accountability. Even soldiers who are politically aligned to a community can end up causing extreme food insecurity. In South Sudan, it has not been the norm for soldiers to receive salaries, even if payrolls have provided important political incentives at certain moments (de Waal, 2019). During the 1990s and 2000s, the rebel SPLA would demand to be fed by the communities in which its forces were based. This involved going house to house to find food. Active chiefs would negotiate with the SPLA to make this food and asset collection predictable and safer for their citizens (Pendle and Anei, 2018). This pattern has continued into recent years. Interviewees also highlighted how soldiers often come to the community when there has been significant insecurity with the justification of increasing security. The community is then forced to provide food to these soldiers. It is this additional need to feed soldiers that can result in hunger reaching catastrophic levels. People cannot refuse to feed soldiers as this would often be met with physical punishment.

While people are acutely aware that soldiers can cause extreme hunger, there is frequently limited space to raise concern within military hierarchies. Open, explicit criticism of warring parties is rarely tolerated. In this context, music has provided a way for communities to complain about soldiers eating their food. The songs often speak of past events and produce a historical memory of these problems. Their contemporary performance is then used to influence the future behaviour of soldiers. The songs are particularly powerful when the soldiers are recruited from the same communities and hence understand the ontologies and moral critiques being invoked.

For instance, the following lines come from a song composed and sung in Jonglei State during the wars of the 1980s; it is still commonly known and sung today. The song speaks of how occupying soldiers in the area were demanding that the community provide cattle for them to eat. A prominent phrase in the song is:

‘*Piny ci riak kɔc a cuet muor*’ – ‘Society has been destroyed because they ate the ox’ (that is, the *muor waar*, the song ox).

On one level, this song is critical of soldiers because they seized assets in a way that left families more food‐insecure. The common Dinka diet includes milk, grains (especially sorghum), fish, and wild fruits, and may also encompass purchased goods such as salt and sugar. Dinka rarely slaughter their own cattle for meat; meat is saved for special occasions such as weddings and funerals. The slaughter or sale of any cattle for meat is assumed to be a sign of desperation. At the same time, the growth of markets and money in South Sudan has increased the trade in cattle (Thomas, 2015; Eliste et al., 2022). In Jonglei and Warrap States, there has been growing export of cattle (for meat) to Juba and to Sudan, and possibly eventually to the Gulf (Duffield and Stockton, 2024). However, there are some assets, such as the *muor waar*, that are not included in these exports.

The song's mention of the *muor waar* made it about much more than material assets. The *muor waar*, also known as the ‘personality ox’, is, as noted above, acquired by a young man, if he and his family can afford it, as he moves from childhood to adulthood, and into a more militarised age group. It is ‘a symbol of his mature identity, cattle wealth, aesthetic esteem and enhanced social status’ (Deng, 1998, p. 108). This ox is a key part of the owner's identity and a display of power and wealth. The young man also then becomes known by the ox's colour or by another common object that shares that colour. Praising the ox becomes synonymous with praising the young man and his whole family and clan. An ox song recorded by Francis Deng highlights the importance of these personality or song oxen to men. The song, recorded by Deng, includes these lines (Deng, 1998, pp. 108–109):

‘My Mijok [*muor* wec] is essential to me,

Like tobacco to a pipe,

When there is no tobacco,

The pipe goes out;

His speed and mine are the same’.

Here, as tobacco is crucial for a pipe not to go out, the *muor waar* is essential for the man's life. A *muor waar* would never usually be killed and might only be given as a gift during a bride wealth exchange. A *muor waar* would only be killed to reprimand the owners for an extremely disgraceful act (Deng, 1973, p. 114).

The soldiers taking of this *muor waar* was not only an insult as it deprived its owner of meat, but also, more significantly, because it undermined the possibilities and status of its owner. The killing of such a *muor waar* was morally shameful; the recalling of this act in song highlights the immoral nature of the soldiers and their failure to act with any care or restraint. Therefore, the song humiliates these soldiers for their past action and serves as a warning against a similar act by soldiers in the future.

In the song, the composer also jokes that they should take the cow bell to chew on. For the composer, the act of chewing on metal is as mad as their killing of the *muor waar*. Their act was not a show of power, but rather a display of madness.

The song goes on to shame the soldiers for suffering and death, but not of people. To speak explicitly of the suffering and death of people caused by such soldiers would probably not have been tolerated politically. Instead, the death and suffering evoked in the song is of the *muor waar* itself. Yet, the ox is equated with people in Dinka cosmological framing (Lienhardt, 1978, p. 17; Deng, 1998, p. 104), making the song a not so well disguised critique of human suffering owing to the actions of the soldiers.

A section of the song that discussed the killing of the *muor waar* can be translated as follows:

‘My Majok [*muor waar*], don't blame me for your death.

I know you are young,

but these are things that happen when the hunger comes,

and our home is in despair’.

The composer asks the ox for forgiveness. It is common in Dinka songs and prayers to address the ox. The composer is clear that the ox died too young. He asks not to be blamed for his death, instead placing the blame on the context of hunger.

The song, implicitly, is requesting that blame for the suffering and death due to famine should not be placed on close individuals in the community. It creates an image of the ox's suffering being beyond his control. The singer is the *muor waar*'s owner and namesake and would usually claim esteem for his careful looking after of the *muor waar*. Yet, the singer is creating a discourse of truth that the context was so abnormal and one of such despair that he should not be blamed. This creates a discourse of emergency and exception, and a period where there should be sympathy for people not being able to uphold fully their usual duties of care. If it can be accepted as true that the time of hunger removed people's ability to stop suffering, then the shame cannot be on those who survived bovine or human suffering.

### 5.2 Songs that create familial shame for famine suffering

In Jonglei and Warrap States, relying on social networks is a crucial way to survive extreme hunger. There is an expectation that people will share the food that they have with extended family members until there is none left (Harragin and Chol, 1998; Maxwell, Gelsdorf, and Santschi, 2012). This is not unique to these regions of South Sudan. Research in Somalia, for example, has also highlighted how people's social connections were instrumental in how well they coped with famine (Maxwell et al., 2016). For instance, many of the famine deaths in Somalia in 2011 were caused by global banking regulations preventing the movement of money within familial networks, stopping family members from being able to keep their relatives alive by sharing resources (Maxwell and Majid, 2016). Social networks can also open up opportunities for some family members to migrate to more food‐secure areas, reducing the food burden on those who remain (Otieno, 2016). At the same time, the importance of social networks cannot be assumed to be static. In many parts of the world, markets, urbanisation, and monetisation have challenged the significance of social networks (Swift, 1993).

In South Sudan, these networks are enforced both socially and legally. For example, previous research shows how the chiefs' courts in Warrap State enforced the redistribution of food among paternal family to the hungriest people, if paternal family members have failed to provide help when they could have done so. It is not only that food is redistributed, but those who failed to give their food voluntarily to needy family members are publicly shamed for their lack of support of persons in need. This enforcing of social networks and the sharing of food saves lives and acts as a safety net for the most vulnerable. At the same time, the public shaming of family members can enforce the idea that they are responsible for preventing famine suffering and, therefore, that if people suffer, the family can be shamed (Pendle, 2023). In the communities where we conducted research, people clearly articulated the causes of famine as being armed conflict and, sometimes, the specific actions of warring parties; however, this was not seen as inconsistent with blaming family members for suffering during famine. It is claimed that social networks, if properly upheld, should be able to prevent extreme suffering even during times of famine.

In songs, we also see familial responsibility to prevent famine suffering being socially enforced. In Jonglei and Warrap States, there were numerous songs that mocked and criticised families for their failure to support their relatives, if there was someone who had died during famine. The songs rebuked them for failing to uphold the strong moral obligation to care for their own.

Furthermore, as songs are a key way in which histories are shared, the narration of familial shame in songs underscores how this can become a part of local histories and last for years and even decades after the famine itself. Our interviewees discussed how these songs would be remembered by future generations, and how they could continue to have tangible implications decades later. For example, families in Jonglei State described being reluctant to allow their daughters to marry into a family that was mentioned critically in a famine song. They underlined how that family's failure to look after their own relatives in the past meant that they could not be certain that their daughter and her children would be properly cared for. This was often used as an excuse to deny someone marriage or to demand a higher bride price.

The following is an example of such a song from Jonglei State. The song shames someone for allowing their mother to die of hunger.

‘Even if you act well off,

we all know that your mother died of hunger,

you should have helped her.

How come you forget so soon,

maybe another hunger is needed to remind you about your mother’.

The song warns the man that even if he now appears to be wealthy, this is not what people will remember about him. Rather, people will always recall that he allowed his mother to die of hunger. Because of this history of failure in relation to his mother's care, acting ‘well off’ is not positive; instead it is morally repugnant, as it makes it appear as if he has forgotten that his mother died. The song reminds him that the wider community has not forgotten and warns people of the long‐term social implications of famine deaths in the family.

Alternatively, people composed songs to praise their own and other clans when there were no hunger deaths. For example, one song from Jonglei State contains the following lines:

‘Keep your money, cows, in this community.

People eat for free.

We don't worry about the hunger.

Didn't they tell you we only hear famine with our ears.

It has not killed anyone from our community’.

This song suggests that this community manages to be hunger‐free and, therefore, others should come to stay and keep their property in this community for safety. It intimates that even if they have heard news of famine from elsewhere, they have not experienced it themselves. The claims imply that proper management of money and cattle within the community allows people to avoid the suffering caused by hunger. This insinuates that communities that do experience famine suffering could have avoided it and are thus responsible, at least in part.

Another song states:

‘No one from our clan has died because of hunger.

You should know our clan has root on the ground.

The famine should also know that.

The clan chief X^3^ is under a strong leadership,

that cannot be shaken by the famine/hunger’.

This song talks about the lack of famine deaths in the clan and suggests that it is their ‘root on the ground’ and, therefore, their actions and character, that prevent hunger deaths. It is something for which the clan can take credit. The success is also attributed to governance within the clan and particularly the strong leadership of the chief. This implies that chiefs have the ability to prevent people from dying of famine if they are ‘strong’.

Another song from Jonglei State praises the chiefs and elders of a certain clan for ensuring that everyone within it had something to eat. The song was by a man who was poor and who people expected not to have the assets to survive famine. He sang:

‘People are asking me how I survived the famine.

Yet, X, Y, Z have all died of hunger during the famine.

Well, I am a son of clan A led by chief B.

People don't die of hunger in his community’.

Importantly, women's songs also praised the clans of their husbands for not allowing them to suffer during famine. It is not uncommon at such times for wives to accuse their husbands of neglecting them, including in the chiefs' courts (Pendle, 2023). Therefore, songs praising a husband's family for famine‐time support sends a public message that it can be trusted to care for the man's wife.

Widows are particularly vulnerable during a famine. Their late husband's family has an obligation to care for the wife and children; however, widows often fear that they do not have priority, especially in difficult times.

The following song was sung by a widow and was about her late husband's family. The praise for famine‐time support honours him:

‘There is nothing more beautiful than having your clan.

We would have not survived this famine.

*Wut* Deng (father of Deng), don't worry,

your children were not forgotten by your clan.

None died of hunger.’

Elsewhere in the song she talks about how the family honoured her husband by reporting what happened to him. Also significant in this song is that the woman underscores how caring for the clan honours the ancestors of the man's family. They are remembered through care for the living.

While these songs record positive stories of communities preventing suffering and death due to hunger, they also reinforce the idea that communities and chiefs can be praised and are, therefore, somehow responsible for the lack of deadly hunger and suffering during famine. Conversely, this implies that communities that failed to prevent famine deaths could have done so.

The extremely deadly nature of some famines since the 1980s in Jonglei and Warrap States, though, means that it is unclear whether families and clans can always prevent famine suffering, however hard they try and however much sacrifice is involved. Families might not be able to keep their fellow relatives alive because all of the food has run out. While food might have been shared when there was some left, famine mortality might have become so high because families collectively and completely ran out of food. It appears that this was a significant dynamic in the large‐scale excess mortality during the Bahr el Ghazal famine in 1988 (Harragin and Chol, 1998).

At the same time, it is not only about all relatives having a lack of food. The armed conflicts that cause the famine, or the associated political decisions, can also interrupt the ability of networks to offer support. For example, families and clans can end up being split up by armed conflict, and they may have limited abilities to send food back to those who remain within a conflict context. This was the case in Somalia in 2011 (Maxwell and Majid, 2016), and it seems to be the case, albeit because of very different restrictions, in Gaza in the Palestinian territories in 2024.

For instance, a young man that we spoke to told us a story about him being in Juba when armed conflict escalated in 2014 in the town of Leer (Unity State) where his family lived. His elderly, widowed mother was left alone in Leer. The conflict cut off access to the town. Food insecurity grew worse over the following years, with a famine eventually declared there in 2017. The man knew that his mother needed support, but, from Juba, he had no way to access the area. And for many years the telephone network in Leer had not been functional. So, he used his social networks to try to access information. Months later a soldier who had passed through Leer brought news to him of his mother's extreme hunger, illness, and inability to move from her home. He was told that she could barely walk anymore. This young man happened to have a brother in the army of senior rank, and they used their political contacts to negotiate individual humanitarian access for his mother to get her to a place of safety, and one with food. Without these networks he would have been unable to access his mother. Many others would have been unable to save their mothers.

The danger, therefore, is that songs that shame families for their members' suffering or deaths during famine might be levying shame on people who had no ability to prevent extreme hunger. Hence, shaming the families misplaces blame. One consequence of this is that people do not want to speak about famine‐time deaths that have occurred within their own family. Throughout the research, we noticed that people always wanted to talk about deaths in other clans and families, even when other sources highlighted that their own histories included famine deaths. People's reluctance to speak about famine suffering and death within their own family reduces their own capability to be politically active vis‐à‐vis famine. This is despite, in other contexts, grief activism by family members being a potentially powerful tool for sociopolitical change (Dominguez, 2003; Hemer, 2010; Stierl, 2016; Al′Uqdah and Adomako, 2018).

### 5.3 Songs shaming those that flee

When there is extreme hunger, people in South Sudan draw on a range of strategies to keep themselves and their communities alive. Alongside social networks, migration has always been a key part of survival. If there is not conflict, during times of hunger, people would migrate to nearby communities or places where there is more access to food. For example, people moved across the borders between Lakes, Unity, and Warrap States to access food in the 1980s. However, in more extreme times of hunger in more recent decades, famines in the Sudans have caused significant migrations over long distances. This was partly because neighbouring communities also did not have food, as in famines like the 1998 *makrup*, and partly because longer distance routes, to places like Khartoum, have become more known and more used as trade and labour migration routes have opened over time. Famine migrations overlapped not only with migrations away from armed conflict, but also migrations to access guns for defence, educational opportunities, and agricultural and military labour (Kindersley, 2017; Akoi and Pendle, 2020).

The famine‐time migration was not without implications for those who remained behind. Communities diminished in size, with local leaders having fewer people to govern. These migrations also had a practical impact on food security among those who stayed, as the loss of labour undermined grain production.

In this final subsection we consider extracts from songs that shame people for fleeing to Khartoum during the famines of the 1980s and 1990s. This happened in part to highlight that people could have survived at home. Flight to the capital of Sudan was presented as a failure, and as a means of escaping social obligations at home to care for their own family members.

A song from Jonglei claims:

‘I cannot escape to Khartoum and leave my family to be eaten by hunger.

Society can have difficult times, but it will eventually get better.

That is why you don't abandon it’.

Songs also sometimes provided an account of survival strategies, implicitly advising people on how they might survive without large‐scale displacement. A famous song from Bahr el Ghazal by Wol Longar about the 1988 famine contains the following lines:

‘I speared an antelope with my sharp spear and refrained from going to Abyei.

I killed an antelope and enjoyed its meat with the vegetable of majong‐thany.

Kurnyuk Panyamum village is endowed with a lot of jamelone [vegetable].

When I move along the *toc* [swampy, grazing areas], I was able to move with ease.

I, together with Mawien Amal, became invincible against hunger,

As we ventured to places of survival in the *toc*.

Some of the vegetables we relied upon included the aruaja, bargo and abiec’.

These lines highlight how it was possible to survive the famine without migrating away from the community, by eating local fruits and vegetables, including those that grow in the *toc*. The song implicitly provides advice on the plants that might be eaten during times of hunger in the future.

The song also includes the following lines:

‘We ate all species and types of different fruits,

hide of the cow

and we could excavate grains from underground storages of ants with hoes.

The hunger hit our civil population’.

All wild fruits were eaten, and animal hides would be soaked in water and rehydrated so that they could provide some nutrition. The comment about ants pertains to how they often take grain and hide it in their hills. People were so desperate for food during the 1988 famine that they would break open the ant hills to try to steal the grains.

The song also talks about the chiefs' courts and their role in keeping people alive:

‘And lastly, there is my fat goat,

which was released with the help of the police officer namely Wol Gak in the house Akot Kuch’.

The final lines of the song then highlight the use of chiefs' courts and police to reclaim loaned livestock to help survive extreme hunger. As discussed elsewhere, during times of hunger, the chiefs' courts actively redistributed resources to those in need (Pendle, 2023). This redistribution through the courts allowed the singer to remain and survive.

Lastly, Wol Longar's song explicitly mentions that famine suffering is caused by laziness. The poor and hungry are undeserving, and should, therefore, join the SPLA:

‘This type of famine does not need a lazy person at all.

You go to Bilpam [SPLA training camps in Ethiopia] to join the army’.

Importantly, the song narrates how possible it was to survive the 1998 famine without fleeing north. It presents a regime of truth in which hard work and local knowledge of the grazing land and courts were adequate to save people from famine suffering and starvation. In this account, those who suffer during famine are represented as being too lazy and, therefore, worthy of shame for their suffering. This is despite the famines of the 1980s and 1990s being caused by the brutal war tactics of the Sudanese government, and the war having prevented people from safely accessing the *toc* (Pendle, 2017). With such high mortality rates in the famine of 1988, it is unclear that people really could have survived by not being lazy and by remaining in the region. The song and its popularity create a regime of truth that potentially masks how difficult famine was to survive.

## 6 CONCLUSION

Deadly contemporary famines have political causes, and so famine will end when it becomes politically unconscionable (de Waal, 2018b, 2024; Conley et al., 2022). In South Sudan, songs and music are a popular source of history and political commentary. They evoke cosmological and normative images and concepts, and communicate powerful, sometimes implicit, messages. Songs provide a means of remembering and retelling, as well as a space for political, public conversations despite the restrictions of war.

Our research in Jonglei and Warrap States shows how common within these songs is a commentary of how people behaved in response to crisis, and which of these behaviours was successful and morally laudable. Soldiers are blamed for violating moral norms and killing a *muor*. At the same time, other songs shame families for allowing famine‐related suffering of their members or criticise those who migrated to safety during famines. They suggest that care and survival despite a lack of migration were possible even when, for many, they clearly were not. Although the majority of the songs that we listened to focused on famines in the 1980s and 1990s, it is still common to hear them today and they influence how people understand responsibility for famine suffering.

If we are to comprehend why famine persists around the world, we need not only to understand international and national politics about famine, but also how famine manifests in the lives of ordinary people who survive. Our focus on songs about famine in South Sudan highlights how important songs can be in recording and communicating local famine politics and shaping understandings of shame and responsibility for famine suffering. Historians of famine have already paid attention to this. Scholars of contemporary famine politics need to explore what more can be learnt by focusing on songs about famine—songs that are being composed at present, and older songs that are being performed and listened to in contemporary times.

Key to ending famine is making it politically reprehensible so that it is avoided by political leaders at all costs (de Waal, 2018a; Conley et al., 2022). Much of this work needs to occur through advocacy and political pressure at a national and international level. At the same time, there is also a need to pay attention to, and potentially to learn from, the politics of famine in communities where famine occurs. Famine scholars and activists need to be aware of the potential for famine songs to create space for political protest and the creation of a politics in which famine is unconscionable. Alternatively, they also need to be cognisant of the powerful role that songs can have in placing blame for famine suffering on families and friends, distracting from a focus on the real causes of famine and muting potential anti‐famine activists.

Music is a particularly useful political tool in many famine contexts. Famines since the 1990s have occurred during armed conflict, and often because of acts of starvation. In times of armed conflict, explicit political debate can be limited, and the subtle messaging in songs becomes a key space for political expression. If international actors really want to stop famine, they should pay careful attention to local, anti‐famine political discourses in famine settings to see how they can support them and ensure that they do not undermine those that push against a politics that tolerates famine. These discourses might also provide material for international advocacy.

Through the example of famine songs in Jonglei and Warrap States, we hope to spark more geographically diverse research in other regions of South Sudan, and other parts of the world, to explore how songs, as well as other forms of artistic expression, shape discourses and opportunities for protest. For instance, in famine‐prone areas of Somalia, there is a strong tradition of poetry; it would be useful to examine similar themes here in relation to poetry. There is also a need to comprehend how discourses in songs, film, and art shape global understandings of famine.

## ETHICS STATEMENT

This paper reports analysis of primary data. The ethics of data collection and analysis were approved by the Research Ethics Committee at the London School of Economics and Political Science and the University of Bath.

## Data Availability

The data that support the findings of this study are available on request from the corresponding author. The data are not publicly available due to privacy or ethical restrictions.

## ENDNOTES

^1^
This paper is based on further analysis after initial findings were presented during a panel titled ‘Famine and Food Insecurity: New Trends and Systems or Politics as usual?’, convened by Susanne Jaspers and Luka Deng Biong, at the International Humanitarian Studies Association's 2023 conference. For more information, see https://conference.ihsa.info/proposed-panels/view/4174/ (last accessed on 16 September 2024).

^2^
South Sudan became an independent country in 2011. Previously it was referred to as Southern Sudan. However, for the purpose of this paper and for ease of clarity for the reader, we refer to this region as South Sudan throughout, even when we are discussing periods prior to 2011.

^3^
The actual names of chiefs and individuals have been removed to preserve their anonymity.
